# Recent Microbial Evolutionary Insights From Metagenomics

**DOI:** 10.1093/gbe/evag029

**Published:** 2026-02-11

**Authors:** Bárbara Moguel, Laura Carrillo Olivas, Mariana G Guerrero-Osornio, Sur Herrera Paredes

**Affiliations:** Chemical and Biological Sciences Department, Universidad de las Américas Puebla (UDLAP), Cholula, Puebla, México; International Laboratory for Human Genome Research, Universidad Nacional Autónoma de México (UNAM), Juriquilla Querétaro, Querétaro, México; Institute of Ecology, Graduate Program in Biological Sciences, Universidad Nacional Autónoma de México (UNAM), Ciudad de México, México; International Laboratory for Human Genome Research, Universidad Nacional Autónoma de México (UNAM), Juriquilla Querétaro, Querétaro, México; Institute of Ecology, Graduate Program in Biological Sciences, Universidad Nacional Autónoma de México (UNAM), Ciudad de México, México; International Laboratory for Human Genome Research, Universidad Nacional Autónoma de México (UNAM), Juriquilla Querétaro, Querétaro, México

**Keywords:** metagenomics, microbial evolution, ancient DNA (aDNA), pathogen evolution, geobiology, microbial adaptation

## Abstract

Microorganisms have profoundly shaped Earth's biological and geological history, from the origins of oxygenic photosynthesis to present-day global biogeochemical cycles. Metagenomics—through its ability to recover genomic information directly from environmental samples—has revolutionized our understanding of microbial evolution by uncovering unbeknownst lineages, revealing functional adaptations, and reshaping our view of the Tree of Life. By bypassing the need for cultivation, shotgun metagenomics and metabarcoding approaches have enabled researchers to investigate microbial diversity, ecology, and evolutionary processes across aquatic, terrestrial, extreme, and host-associated environments. This review highlights recent advances in evolutionary biology driven by metagenomics, including studies on deep evolutionary branching events, microbial adaptation to extreme environments, the evolution of host-associated microbiomes, and the emergence and spread of pathogens and antimicrobial resistance. The integration of ancient DNA has expanded our ability to reconstruct past ecosystems and disease dynamics, offering insights into long-term microbial evolution. In parallel, studies of microbial domestication and urban settings reveal how human practices have shaped microbial genomes over millennia. Despite significant progress, key challenges remain—including improving bioinformatic tools for degraded ancient DNA, resolving deep phylogenetic relationships, identifying adaptive variants, and linking genomic shifts to ecosystem-level processes. The future of microbial evolutionary research will depend on combining longitudinal metagenomic data, experimental evolution, functional assays, and predictive modeling to better understand microbial responses to climate change and anthropogenic pressures. Together, these approaches will deepen our understanding of microbial evolution and its consequences for life on Earth—past, present, and future.

Significance Understanding how microbes evolve is essential for grasping the history and future of life on Earth, yet much of microbial evolution remains hidden because most microbes cannot be grown in the lab. This review highlights how metagenomics—characterizing all genetic material in an ecosystem—has transformed our understanding of microbial evolution. Metagenomic approaches have revealed previously unknown branches of the Tree of Life, uncovered the pathogens behind historical epidemics, and traced microbial adaptation to both human influence and environmental change. Beyond cataloging diversity, metagenomics has also reshaped fundamental biological paradigms, offering new frameworks to understand the origin, dynamics, and evolutionary potential of microbial communities. Metagenomics studies have reinforced the importance of adaptive processes and revealed the extent of microbial horizontal gene transfer and transmission across ecosystems, linking human health to environmental health. Despite this, challenges remain—such as distinguishing adaptive evolution from ecological turnover, resolving complex microbial transmission patterns, and validating molecular mechanisms—that will drive the next generation of metagenomic research.

## Introduction

The study of microbial evolution has traditionally relied on cultivated organisms and—to a lesser degree—fossil records; however, metagenomics has revolutionized our ability to explore previously inaccessible evolutionary events. By studying all the genetic information in a given environment (Handelsman et al. 1998), metagenomics bypasses the need for obtaining pure cultures. Metagenomics has been made possible by synergistic advances in sampling, sequencing, and bioinformatics. Modern metagenomics requires integration of taxonomic compositional data, genetic diversity analysis, computational functional predictions, and spatiotemporal dynamics (Peng et al. 2025). By integrating metagenomic data across ecosystems and timescales, researchers can gain a comprehensive view of microbial evolution, bridging the gap between macroevolutionary (see Glossary) patterns and microevolutionary (see Glossary) mechanisms.

Shotgun metagenomics, attempting to characterize all the genetic information in a sample, allows researchers to reconstruct genomes (West et al. 2018), identify evolutionary significant lineages (Spang et al. 2015), and track evolutionary dynamics at an unprecedented scale (Chase et al. 2021). In parallel, metabarcoding, the amplification and sequencing of genetic markers, allows us to describe the taxonomic diversity in almost any ecosystem. Together, shotgun metagenomics and metabarcoding have led to the discovery of entire new branches in the Tree of Life (Parks et al. 2017), as well as to fundamental insights into the adaptive process, revealing how selection pressures shape microbial genomes in response to environmental shifts (Sunagawa et al. 2015), host interactions (Wolff and Garud 2025), and anthropogenic influences (Danko et al. 2021). Additionally, metagenomics enables the study of evolutionary change over time by leveraging ancient DNA (aDNA) to perform historical environmental and evolutionary reconstructions (Moguel et al. 2021). Metagenomics is also essential for understanding ongoing evolutionary processes, including the emergence of antimicrobial resistance (AMR) (Zhang et al. 2025), adaptation to climate change (Garcia et al. 2020), microbial transmission dynamics (Peimbert and Alcaraz 2023), and the role of horizontal gene transfer (HGT) (*see Glossary*) in shaping microbial diversity (Groussin et al. 2021).

**Fig. 1.**
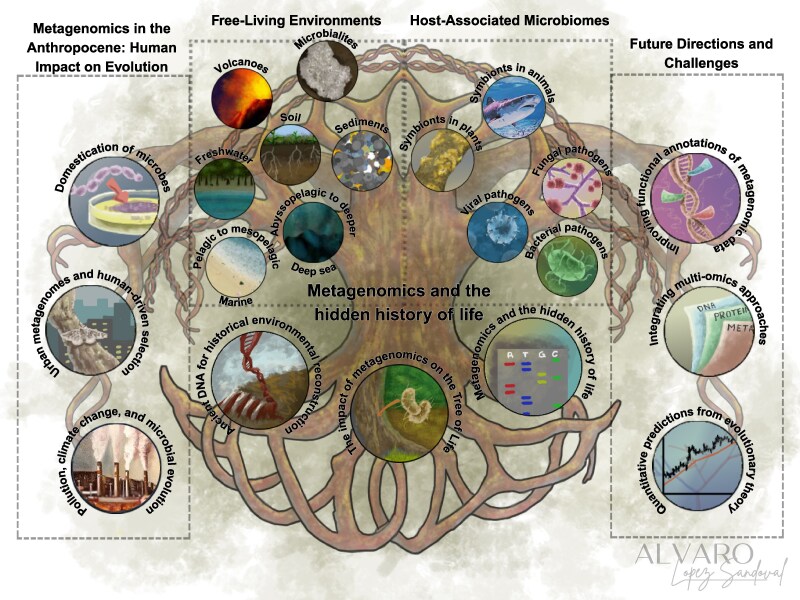
Metagenomics and the hidden history of life. Metagenomic approaches uncover microbial diversity and evolutionary processes across diverse environments. The figure is organized into four thematic sections. Center left: Free-living environments, highlighting microbial diversity and evolutionary processes in volcanoes, soils, sediments, freshwater, marine, and deep-sea habitats. Center right: Host-associated microbiomes, showcasing microbes in various hosts and the role of microbial and viral pathogens. Left: Metagenomics in the Anthropocene, emphasizing human impacts on evolution through domestication, urbanization, and climate change. Right: Future directions and challenges, focusing on advances in functional annotation, integration of multi-omics approaches, and quantitative evolutionary predictions. Branching connections represent relationships that can be studied by metagenomics. Together, the figure illustrates how metagenomics reshapes our understanding of microbial evolution, ecosystem function, and the complex interactions between microbes, hosts, and human activities. Illustration by Álvaro López-Sandoval.

In this review, we highlight key areas where metagenomics has provided novel evolutionary insights—many of which would have been impossible to achieve using pure culture based approaches. Metagenomic investigations have contributed insights across nearly the entire span of Earth's geological history, encompassing aquatic, terrestrial, extreme, host-associated, and urban ecosystems. We conclude by outlining major open questions and promising directions for future research (Fig. 1).

## Refining the Tree of Life

The origins of metagenomics are deeply intertwined with the study of the Tree of Life. Early efforts played a crucial role in confirming the three-domain model of life, leading the microbiologist Carl Woese to predict that sequencing's “most profound and lasting effect will be on our perception of evolution and its relationship to the rest of biology” (Woese 1987). During the 1990s and early 2000s, it became evident that only a tiny fraction of microbial diversity had been identified. One landmark discovery was the identification and later cultivation of the bacteria *Candidatus Pelagibacter communis*, the most abundant organism in the ocean (Rappé et al. 2002). Genomic analyses of *Ca. P. communis* revealed an extremely small genome (1.3 Mbp), reinforcing the hypothesis that genome streamlining optimizes energy efficiency and facilitates dominance in nutrient-limited marine environments (Giovannoni et al. 2005).

Since then, the study of superabundant microbes (SAMs) has expanded dramatically, raising fundamental questions in molecular evolution. The astronomical population sizes of these microbes imply that every possible mutation happens many times every generation, challenging the interpretation of standard phylogenetic and population genetic analysis. A central issue concerns the discrepancy between the observed levels of nucleotide diversity in SAMs (e.g. *Prochlorococcus*, *Gephyrocapsa huxleyi*) and the genetic diversity predicted by population genetics theory (Filatov and Kirkpatrick 2024). This mismatch has led to the proposal that the quasi-neutral model, which dominates contemporary evolutionary thinking, may not adequately describe evolution in SAMs. Instead, their evolutionary dynamics may be more consistent with the classical panselectionist (*see Glossary*) framework (Filatov and Kirkpatrick 2024).

Over time, the advent of massively parallel sequencing and the development of tools to recover metagenome-assembled genomes (MAGs) (*see Glossary*) transformed our perspective of global biodiversity. In the context of the Tree of Life, the accumulation of MAGs challenged the view that the three domains of life—Bacteria, Archaea, and Eukarya—contribute equally to biodiversity. Instead, metagenomic data suggest that bacteria comprise the largest fraction of known biological diversity (Hug et al. 2016). However, the precise fraction depends on the marker genes used for phylogenetic reconstruction (Parks et al. 2017), and the choice of markers remains an ongoing debate (Moody et al. 2022), with some studies using different sets of markers for different domains (Parks et al. 2017), while others use a single set of universal markers (Hug et al. 2016; Moody et al. 2022).

Shotgun metagenomics has further evidenced the close evolutionary relationship between archaea and eukaryotes (Hug et al. 2016), shedding light on the origin of this later group. The discovery of Asgard archaea from reconstructed deep-sea MAGs revealed that these organisms harbor a number of eukaryotic signature genes involved in membrane remodeling and vesicular trafficking, indicating that cellular complexity started to evolve before the divergence of eukaryotes and archaea (Spang et al. 2015). Despite this, within the eukaryotic domain, metagenomics has been less widely applied, partially because of the challenges of assembling larger and more complex genomes. Nonetheless, recent studies have demonstrated the feasibility of recovering eukaryotic MAGs using *k*-mer analysis and marker sets to assess completeness and contamination (West et al. 2018). The application of these techniques at scale has revealed eukaryotic MAGs without closely related genomes (West et al. 2018), suggesting that these approaches will expand our understanding about diversity and evolution along the eukaryotic branch of the Tree of Life.

Beyond phylogenetic reconstructions, shotgun metagenomics has enabled functional inferences throughout the Tree of Life. Particularly striking was the observation that deep branching lineages in both bacteria (the Candidate Phyla Radiation or CPR) and archaea (the DPANN kingdom, named after the first five groups that comprised it: Diapherotrites, Parvarchaeota, Aenigmarchaeota, Nanoarchaeota, and Nanohalarchaeota) are characterized by reduced genomes, typical of obligate symbiotic lifestyles, suggesting an ancient evolutionary transition toward obligate symbiosis occurred independently in two domains of life (Castelle et al. 2018). Due to their obligate symbiotic nature, CPR and DPANN microbes have proven difficult to culture; moreover, the presence of introns in their 16S rRNA genes (Brown et al. 2015) obscures their presence in standard metabarcoding approaches. As a result, shotgun metagenomics remains the primary method for identifying and characterizing these lineages.

## Reconstructing Past Environments

Over the past two decades, research on aDNA has advanced substantially, ushering in a new era of studies focused on reconstructing past environments. Notably, the recovery of plant and animal aDNA from permafrost cores—dating back between 10,000 and 400,000 years ago—has revealed major shifts in the taxonomic composition and diversity of Beringian vegetation and fauna. When dealing with aDNA, it is key to address the contamination risks during sample collection and laboratory processing, and environmental aDNA presents additional complexities, requiring more stringent workflows to ensure the recovery of DNA in both sufficient quantity and quality for downstream analysis (see Box 1). To complement standard approaches, methods based on *k*-mer similarity have been proposed to infer the geographic origins of ancient samples (Bozzi et al. 2024). These methods cluster samples based on shared genomic content and spatial proximity, offering novel insights into the biogeography of historical microbial communities (Bozzi et al. 2024).

Box 1. Ancient metagenomics pipelines Metagenome-assembled genomes (MAGs) are a cornerstone of modern metagenomic analysis. Recovering MAGs from aDNA is especially difficult due to the highly fragmented nature of ancient DNA and the elevated risk of contamination from modern environmental microbes, both of which can obscure the original composition of ancient microbial communities. As a result, specialized procedures that take these considerations into account are required in both molecular experiments and computational analyses.

The first step of the analysis of ancient pathogens involves the QC of the sequencing reads, including the removal of reads and filtering out low quality and short (e.g. <30 bp) fragments (Renaud et al. 2019; Lien et al. 2023). While new tools have emerged for this purpose, adapter removal is widely used in the field due to its efficiency and accuracy, but new tools like leeHom are promising (Renaud et al. 2014; Lien et al. 2023).

The choice of the taxonomic software used in microbiome and pathogen identification is also a critical step (Orlando et al. 2021; Malyarchuk et al. 2022). Mapping against single reference genomes can introduce bias, especially when working with short sequences that carry genetic variants (Orlando et al. 2021). Different classification methods exists, such as those employed by MetaPhlAn, Kraken, and de novo genome assembly, and differ in the way they calculate species abundance (Li et al. 2015; Nurk et al. 2017; Breitwieser et al. 2018; Lu et al. 2022; Malyarchuk et al. 2022; Blanco-Míguez et al. 2023). New pipelines as aMeta, HAYSTAC, HOPS, Mapache, nf-core/mag, nf-core/eager, and nf-core//taxprofiler have recently gained recognition (Herbig et al. 2016; Hübler et al. 2019; Ewels et al. 2020; Yates et al. 2021; Dimopoulos et al. 2022; Krakau et al. 2022; Neuenschwander et al. 2023; Pochon et al. 2023; Stamouli et al. 2023).

Moreover, there is a growing emphasis on reducing false positives in microbiome and ancient pathogen identification (Bos et al. 2019; Peyrégne and Prüfer 2020; Dimopoulos et al. 2022; Neuenschwander et al. 2023; Pochon et al. 2023). These approaches are not only efficient in terms of memory and time but also provide a reliable means of distinguishing genuine pathogens in each sample.

Despite challenges, aDNA metagenomics has emerged as a powerful tool for reconstructing past ecosystems and their transformations over time (Nguyen et al. 2023b). A study in Northern Siberia examined 6,700-year-old sedimentary records, revealing significant ecological transitions from boreal forests to tundra ecosystems and documenting their impact on microbial communities (Perfumo et al. 2023). Another study analyzed ancient feces dating back 1,000 to 2,000 years from southwestern United States and Mexico, reconstructing 498 medium- and high-quality ancient MAGs. Notably, 39% of the 181 ancient MAGs represented previously undescribed species-level genome bins (Wibowo et al. 2021). Moreover, research on coprolites from extinct megafauna has demonstrated the potential to identify host species, reconstruct diets, and detect associated parasites, offering an integrated view of past animal–environment interactions (Petrigh et al. 2021).

This approach has also been applied to tropical sediment cores, such as those recovered from Lake Chalco in central Mexico. Spanning the last 12,000 years, corresponding to the Holocene, the integration of taxonomic and functional gene analyses with diatom fossil records and geochemical data revealed substantial changes in both the surrounding landscape and the lake's ecosystem. Notably, signals of human impact were detected around 6,000 years ago, indicating anthropogenic alterations to the environment (Moguel et al. 2021).

Sedimentary aDNA allows researchers to obtain rich genomic information without relying on preserved macrofossils or invasive excavation. At the same time, sedimentary aDNA microbial signatures can be obscured by modern microbial activity. Despite this, a groundbreaking example comes from northern Greenland, where sediment samples preserved in permafrost have yielded the oldest known DNA—approximately 2 million years old. The original analysis revealed traces of over 100 animal and plant species, indicating that this now-frozen region once supported a lush, more temperate ecosystem (Kjær et al. 2022). Interestingly, a subsequent analysis of the same data used DNA damage estimation to stratify microbial aDNA reads and found signatures of methanogenic archaea, consistent with the elevated temperature of the time (Fernandez-Guerra et al. 2025).

## Selective Pressures in Aquatic Environments

Aquatic environments represent a key frontier for biological research—offering insights into the history of life and promising biotechnological applications. Metagenomic studies have shown that oceanic ecosystems—especially deep-sea and hydrothermal vent communities—are highly diverse and include novel taxonomic groups with a highly specialized metabolism that is dependent on metabolic exchange among community members (Zhou et al. 2022). One possible mechanism supporting this vast microbial diversity is the formation of biofilm structures, which create protected microenvironments where rare species can persist, exchange genetic material, and accumulate genetic innovations. This dynamic allows the maintenance of a genetic reservoir that can be mobilized in response to ecological perturbations (Wang et al. 2017).

At the global ocean scale, early metabarcoding surveys found that temperature was the strongest factor associated with community composition, leading the authors to conclude that dispersal plays a subordinate role to temperature in shaping taxonomic and functional diversity in the ocean surface (Sunagawa et al. 2015). These results have been reinforced by shotgun metagenomics over the years (Sunagawa et al. 2020). At the molecular level, assembly-free shotgun metagenomic analysis of hundreds of globally distributed samples, up to 10,500 m deep, revealed that temperature, and not nutrient limitation or depth, explains bacterial and archaeal genome size variation (Ngugi et al. 2023). This genome size variation was mostly explained by gene elongation and duplications leading to larger genomes in cooler temperatures (Ngugi et al. 2023). Given that similar trends have been observed in freshwater (Lear et al. 2017) and soils (Sorensen et al. 2018), the selection for small genomes at high temperature might turn out to be one of the primary consequences of global warming, as elevated mutation rates at high temperatures (Waldvogel and Pfenninger 2021 ) make it more costly to maintain a large genome (Ngugi et al. 2023).

Nutrient limitation is another major environmental stressor that shapes microbial evolution in nutrient-poor oligotrophic (*see Glossary*) aquatic environments. This evolutionary pressure often leads to genome streamlining—a process that reduces metabolic cost by conserving resources and enhancing fitness (Giovannoni et al. 2014). Genome-resolved metagenomics in freshwater lake ecosystems has shown that this evolutionary trajectory is reflected in reduced GC content, fewer sigma factor-encoding genes, higher coding density, lower gene redundancy, and functional shifts in pathways such as cell motility and ATP-binding cassette (ABC) transporters (Shah et al. 2024).

Rivers are unique among aquatic environments because their longitudinal nature exposes them to diverse climates, ecosystems, pollutants, cultural practices, and biodiversity. Additionally, rivers act as major conduits of dissolved organic matter (DOM), transporting vast amounts from terrestrial basins and into the ocean. Recently, meta-metabolomics coupled with shotgun metagenomics across seven northern hemisphere rivers confirmed a tight link between DOM composition and microbial community, while also highlighting significant remaining river-to-river variation (Danczak et al. 2024). Ongoing large-scale efforts to characterize the metagenomes of some of the world's largest rivers (Rout et al. 2024b; Ren et al. 2025) provide a unique opportunity to explore microbial adaptation and evolutionary processes in these dynamic ecosystems.

## Microbial Evolution in Extreme Environments

**Fig. 2.**
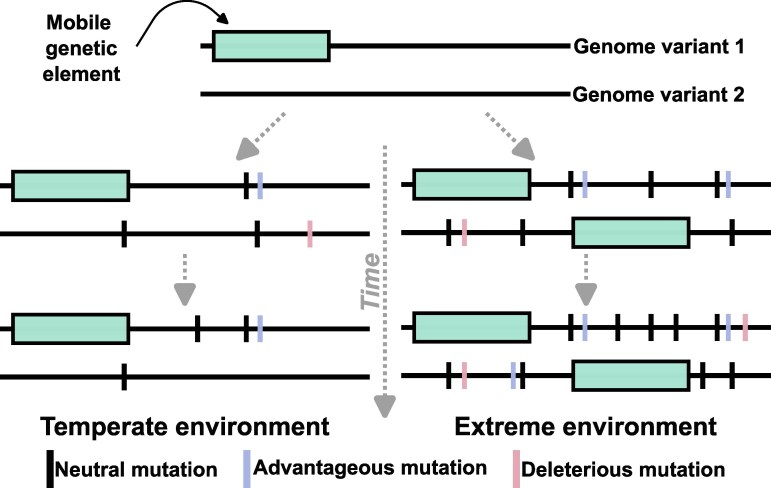
Genomic evolution scenarios in temperate versus extreme environments. The figure illustrates potential evolutionary trajectories of a hypothetical population that begins with two genomic variants (horizontal lines) and evolves over time (dashed arrows) in either a temperate (left) or extreme (right) environment. Left: In a temperate environment, mutations arise gradually. Advantageous variants (purple) increase in frequency through positive selection, deleterious mutations (pink) are purged from the population, and neutral mutations (black) accumulate slowly. Right: In an extreme environment, elevated mutation rates, increased horizontal gene transfer, and stronger positive selection accelerate genomic divergence. Mutations accumulate more rapidly, and linked neutral and deleterious mutations can hitchhike to higher frequencies. Enhanced activity of mobile genetic elements (cyan) further contributes to divergence from the ancestral genomes. Together, the figure illustrates how extreme environments can amplify evolutionary rates and mechanisms, leading to distinct evolutionary outcomes.

Comparative studies reveal that microorganisms inhabiting extreme aquatic environments—such as acid mine drainage sites, saline lakes, and hot springs—exhibit more pronounced evolutionary patterns than those in more temperate settings (Fig. 2), including both elevated relative evolutionary rates (rERs; *see Glossary*) (Li et al. 2014) indicative of adaptive evolution, as well as strong purifying selection for targeted metabolic genes (Rojas et al. 2024). This pattern is thought to result from a combination of stress-induced mutation rates, strong specific selective pressures, increased positive selection in core genes, and a higher abundance of mobile genetic elements (Fig. 2) (Li et al. 2014; Rojas et al. 2024).

Additionally, metabarcoding has revealed a high diversity of benthic foraminifera associated with methane seeps in the Arctic (Nguyen et al. 2023a). Complementary metagenomic and metatranscriptomic analyses of archaeal and bacterial communities in the hydrothermally heated sediments of the Guaymas Basin have elucidated microbial adaptations to temperature and chemical gradients (Mara et al. 2023). Another noteworthy example is microbial adaptation to heavy metals; in acidic, mercury-rich warm springs of the Ngawha Geothermal Field in New Zealand, genome-resolved metagenomics coupled with geochemical analyses uncovered acidophilic and mesophilic sulfur- and iron-cycling bacteria, mercury- and arsenic-resistant bacteria, as well as thermophilic and acidophilic archaea (Gionfriddo et al. 2020). Overall, these studies demonstrate the widespread capacity for microbial adaptation to extreme conditions.

Studies in the Cuatro Ciénegas Basin (CCB) in Northern Mexico—an oligotrophic oasis—reveal unique microbial adaptations to nutrient limitation within microbial mats. Furthermore, the CCB serves as a valuable model for understanding early life and astrobiology. Experimental evidence suggests that reduced phosphorus species, likely delivered by meteoritic impacts, played a critical role in prebiotic chemistry on early Earth (Tapia-Torres and Olmedo-Álvarez 2018). Modern analogs of these conditions persist in the CCB, where phosphorus-limited waters and microbial communities reflect geochemical constraints thought to have shaped early biogeochemical cycles (Souza et al. 2018).

Microbial mats are dense, stratified communities of microorganisms with ancient origins and remarkable ecological adaptability. Certain microbial mats facilitate carbonate precipitation, leading to the formation of microbialites. The evolutionary significance of microbialites lies in their role as ancient, community-driven structures shaped by metabolic cooperation and circadian cycling—processes that have persisted for 3.7 billion years (Nutman et al. 2016). Microbialite-forming mats, particularly those dominated by cyanobacteria, pioneered oxygenic photosynthesis; this pivotal transformation facilitated the emergence of aerobic metabolisms, eukaryotes, and multicellular life. By revealing how early microbial communities organized, adapted, and altered global geochemical cycles, microbialite studies offer a unique window into primordial ecosystems, shedding light on the ecological innovations that laid the foundation for complex life and major evolutionary transitions on Earth (Vignale et al. 2022).

Hypersaline lakes serve as natural laboratories for studying the evolution of microbialite systems. In the maar lake (*see Glossary*), Rincón de Parangueo metabarcoding analyses have revealed a microbial community dominated by halophilic Archaea and sulfur-cycling bacteria (Sánchez-Sánchez et al. 2021, 2023). Shotgun metagenomics has further linked carbonate precipitation to microbial activity along high salinity and alkalinity gradients (Saghaï et al. 2015). In Australian saline lakes, microbialite-forming mats exhibit a conserved functional core of critical pathways, such as oxygenic photosynthesis, which are conserved across these geographically and taxonomically distinct microbial communities (Warden et al. 2016; Nguyen et al. 2022). This functional conservation underscores the central role of photosynthesis in generating alkaline microenvironments through CO₂ uptake, which promotes calcium carbonate supersaturation (Nguyen et al. 2022). Notably, microbialite systems also display robust viral defense mechanisms, including CRISPR/Cas arrays, reflecting intense phage pressure in these densely populated habitats (Suosaari et al. 2016). Similar processes occur in Shark Bay, Australia stromatolites, where Cyanobacteria form the architectural backbone by elevating pH through photosynthesis. Their syntrophic interactions with sulfate-reducing and heterotrophic bacteria create a diel lithification cycle (Campbell et al. 2020; Nguyen et al. 2022; Skoog et al. 2022). This daily coordination between autotrophs and heterotrophs exemplifies how microbial interactions transcend phylogenetic boundaries to sustain carbonate mineralization under hypersaline conditions.

Subsurface microbial communities along the Central American Volcanic Arc (CAVA) are strongly influenced by tectonic processes. Metagenomic and geochemical analyses reveal that factors such as slab depth, rock type, and hydrothermal fluid maturity have a greater impact on microbial community structure than geographic location (Basili et al. 2024). This underscores the dominant role of geological controls in shaping microbial ecology in these environments (Basili et al. 2024). Additionally, mesocosm studies investigating microbial sulfur cycling in active nickel and copper mine tailings demonstrate that biotic processes can significantly influence acid generation and sulfur transformations, often deviating from abiotic predictions (Gordon et al. 2024).

## Microbial Transmission Across Hosts

**Fig. 3.**
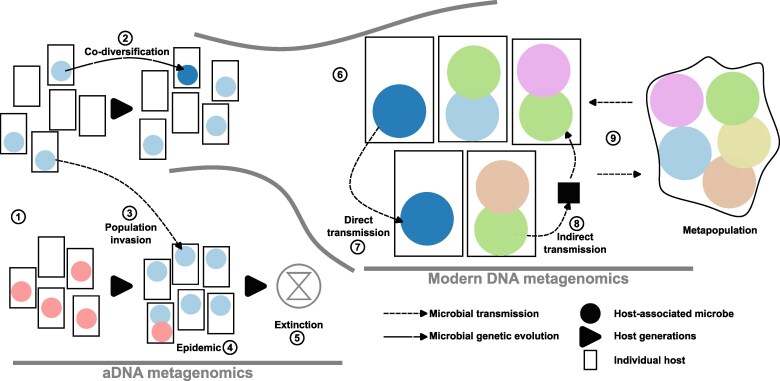
Population dynamics of host-associated microbes across evolutionary timescales. Metagenomic analyses of modern and ancient DNA provide complementary insights into microbial evolutionary processes operating within and between host populations over different timescales. From ancestral host populations (1), each harboring a distinct resident microbiota (indicated by different colors), microbial variants can evolve and be transmitted across host generations within the same population, leading to host–microbe co-diversification (2). Alternatively, microbes from one host population may invade another host population (3), where they can displace endemic microbiota and give rise to epidemics (4). In extreme cases, such outbreaks may contribute to the extinction of the invaded host population (5). Ancient DNA (aDNA) metagenomics enables the direct observation of these processes. In contemporary host populations (6), metagenomic approaches allow the disentangling direct (7), and indirect (8) transmission dynamics, as well as metapopulation-level transmission potentially involving other species (9).

At shorter timescales, the invasion and persistence of microbial species within a host is a microevolutionary process (Herrera Paredes and Lebeis 2016). Microbial dispersal and host-to-host transmission are key factors influencing the evolutionary dynamics of host-associated microbiota (Fig. 3). Host-microbiota systems can be broadly categorized as open, closed, or mixed symbioses, depending on the relative contributions of horizontal and vertical transmission of microbial lineages between hosts (Perreau and Moran 2021 Aug 13). While open symbiosis is generally expected to reduce the potential of host-microbiota coevolution, theoretical work has shown that even under fully open transmission, when microbial fitness is tied to host fitness, selection on colonized hosts can influence allele frequency trajectories and generate nonstandard patterns of genetic diversity, distinguishing host-microbiota evolution from single-species population dynamics (Roughgarden 2023).

The microbiotas across the human body have served as a valuable model for studying these dispersal and transmission dynamics. A recent study, using shotgun metagenomics and high-throughput culturomics of *Cutibacterium acnes* and *Staphylococcus epidermidis* from the facial skin microbiota, revealed that neutral processes—such as priority effects (*see Glossary*)—play a critical role in the success of bacterial lineages and genotypes (Baker et al. 2025). These priority effects likely result in significant barriers to host-to-host transmission, resulting in a pattern where individuals harbor unique strain assemblages, and strains are shared within families but not between unrelated individuals (Baker et al. 2025). Interestingly, *S. epidermidis* exhibits higher strain turnover than *C. acnes*, which correlates with a larger and more dynamic accessory genome. This suggests that adaptive processes are relatively stronger in *S. epidermidis*, driving greater genetic variation, potentially mediated by competitive interactions and HGT (Baker et al. 2025). Complementing this, the creation of the Vaginal Microbial Genome Collection (VMGC), which comprises over 33,000 genomes, uncovered specific microbial functions associated with vaginal health (Huang et al. 2024).

Within the human gut microbiome, shotgun metagenomics has been used to reconstruct the site frequency spectrum (SFS; *see Glossary*) of prevalent gut bacteria, revealing a pattern consistent with an oligo-colonization model (*see Glossary*), in which only a few distinct strains establish within each individual (Garud et al. 2019). An important practical corollary of this observation is many gut microbiome samples, in which a single dominant strain of a species is present, can be “quasi-phased” such that pairs of alleles can be assigned to the same haplotype (*see Glossary*) with high confidence, and linkage disequilibrium (LD; *see Glossary*) can be computed between these pairs of alleles (Garud et al. 2019). While oligo-colonization implies some level of local adaptation, selection scans in bacterial genomes are complicated by fluctuating demographics and variable recombination rates. Recently, a statistic called the integrated Linkage Disequilibrium Score (iLDS) has been proposed; this statistic identifies genomic regions with higher LD between in non-synonymous than synonymous sites (likely reflecting hitch-hiking around a recent selective sweep) and elevated LD relative to the genomic background (to detect recombination-mediated sweeps) (Wolff and Garud 2025). Applying this method to global shotgun metagenomic datasets, researchers detected 309 selective sweeps across 16 prevalent gut bacterial species in 24 human populations. The majority of these sweeps were unique to individual populations, but there was greater sharing of adaptive alleles among Westernized populations, suggesting common selective pressures in industrialized environments. Notably, these sweeps were enriched in genes related to carbohydrate metabolism, reinforcing the idea that host diet is a key driver of bacterial evolution in the gut (Wolff and Garud 2025). Moreover, these findings are consistent with prior evidence that HGT rates are higher in the gut microbiomes of Westernized populations (Groussin et al. 2021). Another study used phylogenetic distances, after excluding HGT events, revealing clonal sweeps among gut commensal bacteria that span decades and distribute globally (Yu et al. 2024). These longer-term sweeps were enriched for genes involved in the modification of surface glycans and glycoconjugates known to interact with the host immune system (Yu et al. 2024). Overall, these studies indicate that microbial adaptation to the human host happens at different scales (Fig. 3), with HGT dominating in the shorter—within population—timescales and clonal sweeps having a stronger impact in the years-to-decades—between population—scales.

In urban settings, transmission dynamics might be mediated by indoor surfaces and dust particles (Hartmann et al. 2016; Ding et al. 2020; Maamar et al. 2020) (Fig. 3). Studies of subway systems across multiple cities have consistently found species from the human skin microbiome (Afshinnekoo et al. 2015; Hsu et al. 2016; Kang et al. 2018; Danko et al. 2021; Leung et al. 2021; Wu et al. 2022), suggesting that these environments may play a role in microbial transmission between individuals. However, systematic evaluations of paired human and environmental samples are still missing, making it difficult to determine how frequently microbes are exchanged between hosts and urban surfaces and what implications this may have for public health (Peimbert and Alcaraz 2023). For example, a recent large metagenomic found very few instances of transmission between patients that shared time and space in a single hospital (Siranosian et al. 2022).

Among plant hosts, shotgun metagenomic sequencing presents unique challenges due to the difficulty in separating microbial DNA from host-derived DNA. Several strategies are used to enrich for the microbial fraction, including physical separation of microbial cells prior to DNA extraction (Masuda et al. 2024), DNA capture-based methods (Lohmaneeratana et al. 2024), and culture enrichment approaches (Lundberg et al. 2022; Baker et al. 2025). These techniques have facilitated identification of host-associated bacterial and fungal communities. For example, physical separation of intact bacterial cells, followed by enzymatic digestion of free DNA, and long-read sequencing allowed the recovery of plasmids and MAGs from the previously recalcitrant lineage *Candidatus Saccharibacteria* in the rice phyllosphere (Masuda et al. 2024). Other physical separation approaches have been used to reveal functional shifts in root-associated microbial communities under environmental stress, suggesting that microbial functional differences may contribute to plant resilience (Hernández-Álvarez et al. 2022). If these functional shifts result from adaptive changes in microbial populations, they could imply coevolutionary processes between plants and their microbiota. However, these results remain correlative, and population genetic approaches will be necessary to disentangle selection from ecological processes and establish the evolutionary significance of these patterns.

Parallel to the work on the human facial skin microbiome (Baker et al. 2025), high-throughput culturomics has proven key to understanding the transmission dynamics of plant-associated microbiota. In a recent work, culture enrichment of *Pseudomonas* and *Sphingomonas*, followed by shotgun metagenomic sequencing, was used to characterize the genomic diversity of these bacterial taxa within and between individual Arabidopsis plants across fields and seasons. Results suggest that microbial transmission occurs more frequently between neighboring plants than from soil reservoirs, indicating that local microbial dispersal plays a dominant role in shaping plant leaf microbiomes (Lundberg et al. 2022).

Culture enrichment approaches have also been applied to animal associated microbiomes, such as in the *Aliivibrio*-*Euprymna* symbiosis. A decade-long study (∼15,000 to 20,000 bacterial generations) revealed that competitive dominance is insufficient to purge bacterial diversity. Instead, recurrent secondary colonization of the squid (*Euprymna*) host by distinct *Aliivibrio* haplotypes suggests that external environmental factors interact host-imposed selective pressures to maintain bacterial diversity over long timescales (Soto et al. 2012), though the targets of these selective pressures remain unknown. Overall, multiple lines of evidence emphasize the role of external microbial reservoirs in structuring the genomic diversity of host-associated microbes in open and mixed symbiosis (Soto et al. 2012; Lundberg et al. 2022; Roughgarden 2023).

## Long-Term Host–Microbe Coevolution

Given the importance of external reservoirs (Soto et al. 2012; Lundberg et al. 2022; Roughgarden 2023), environmental factors (Soto et al. 2012; Hernández-Álvarez et al. 2022), and host lifestyles (Wolff and Garud 2025) in shaping host-associated microbiomes, a key question is whether long-term coevolution between hosts and microbes is occurring and how to distinguish true coevolution from environmental sorting and microbe–microbe interactions. To address this, large-scale comparative analyses of metabarcoding datasets have been employed. One such study analyzed 4,000 publicly available 16S rRNA amplicon samples from insect microbiomes, spanning 246 studies. Interestingly, the analysis revealed that host species identity was the strongest factor determining both microbiome composition and diversity, while host ecological factors had a comparatively minor effect. However, the study also found that while host phylogeny was statistically significant, it had weak explanatory power in determining microbiome structure (Malacrinò 2022). These findings suggest that, if host-microbiome coevolution occurs, it may operate on timescales shorter than macroevolutionary host divergence. Consistent with this idea, phylosymbiosis (*see Glossary*)—the pattern where microbial community similarity mirrors host phylogenetic relatedness—has been observed in narrower clades of both mammals and insects (Brooks et al. 2016), suggesting that host-associated microbiomes can exhibit evolutionary stability at shorter evolutionary timescales.

Beyond metabarcoding, comparative shotgun metagenomics has been used to explore long-term coevolutionary dynamics. One such study examined the dental calculus microbiota from ancient samples across diverse mammalian species, reconstructing ancient MAGs and identifying functional signatures across hosts. Despite taxonomic variability, core metabolic processes were highly conserved (Brealey et al. 2020). However, the extent to which these findings reflect true functional conservation versus annotation biases in metagenomic pipelines remains uncertain. Notably, the study also detected putative oral pathogens and ancient AMR genes, demonstrating that many AMR mechanisms were already present in animal microbiomes long before mass antibiotic production (Brealey et al. 2020). While the selective advantage of these resistance genes in ancient microbial communities remains unclear, their widespread presence suggests that they are likely shaped by environmental and microbial interactions rather than host or anthropogenic pressures.

## Ancient Pathogen Evolution

Although only a fraction of the host microbiome causes disease, cellular and viral pathogens exert disproportionate pressure on host evolution. Unlike commensals (*see Glossary*), pathogens face intense selection from immune defenses, driving coevolutionary arms races that shape health and population genetics at different scales (Fig. 3). Understanding these dynamics requires tracking pathogen evolution through time—an increasingly feasible task thanks to aDNA metagenomics (Spyrou et al. 2019; Orlando et al. 2021; Malyarchuk et al. 2022; Ávila-Arcos et al. 2023).

Recently, human pathogens like *Yersinia pestis*, *Mycobacterium leprae*, *Salmonella enterica*, and hepatitis B virus have become focal points (Yates et al. 2025). Challenges remain in authenticating aDNA, managing contamination, and interpreting functional data (see Box 1). Nowadays, integrating information from virulence factors (*see Glossary*) and other lines of evidence is essential for contextualizing past epidemics.

Ancient *Y. pestis* MAGs trace its emergence from the enteric pathogen *Y. pseudotuberculosis* via gene loss and horizontal transfer, leading to flea-borne transmission and pandemic potential (Demeure et al. 2019; Neumann et al. 2022; Eaton et al. 2023). Two broad clades emerge: one associated with historic pandemics such as the Justinianic Plague (∼1,500 to 1,300 years ago) and the Black Death (∼675 years ago) and an earlier lineage—the Late Neolithic and Bronze Age (LNBA) clade—circulating ∼6,400 to 2,700 years ago (Susat et al. 2024).

LNBA strains lacked key virulence factors for flea-borne spread, suggesting alternate transmission modes (Andrades Valtueña et al. 2022; Swali et al. 2023). Of these, the *pla* gene, which encodes a plasminogen activator protease responsible for virulence and pathogenicity in bubonic and pneumonic plague models, has been critical in their evolution, with evidence that changes in its copy number modulate virulence and transmission dynamics (Sidhu et al. 2025). Genomic evidence places these strains across Europe and Asia, including Britain ∼4,000 years ago (Spyrou et al. 2022; Swali et al. 2023). The Second Pandemic (∼675 to 225 years ago) is mostly linked to a single lineage, indicating long-term persistence, though local diversification and reintroductions also occurred (Clavel et al. 2023; Eaton et al. 2023; Parker et al. 2023).

Coinfections of *Y. pestis* with *Treponema pallidum pertenue* and *Haemophilus influenzae* reveal complex disease burdens (Giffin et al. 2020; Guellil et al. 2022a). Intrafamilial transmission highlights the social and spatial dimensions of outbreaks (Kılınç et al. 2021). Genomic variability in strains from ∼400 years ago (Second Pandemic) highlights differences in virulence and host interaction (Seguin-Orlando et al. 2021). These findings underscore *Y. pestis*’ adaptability, with plasmids and pathogenicity islands playing key roles (Bonczarowska et al. 2023; Pitta et al. 2023; Susat et al. 2024).

Ancient *S. enterica* MAGs provide insight into the long-term evolution of virulence. Strains dating back 6,500 years already carried type III secretion effectors and virulence plasmids (Key et al. 2020; de-Dios et al. 2021). Comparative work on *S. Paratyphi C* reveals co-diversification between host (Fig. 3) and *Salmonella's* pathogenicity island 7, suggesting local adaptation and selective pressures (Zhou et al. 2018; de-Dios et al. 2021; Wu et al. 2021; Neumann et al. 2022). These results point to long-term conservation of core virulence mechanisms, while also revealing genomic fluidity driven by ecological and host factors.

Despite its highly degraded genome and in vitro culture challenges, *M. leprae* shows remarkable genomic conservation (Mi et al. 2024). Yet, ancient MAGs of *M. leprae* reveal a broader distribution of lineages than previously thought. Branches previously considered geographically restricted have been identified in early medieval Europe (Pfrengle et al. 2021). At Winchester's leprosarium, several lineages of *M. leprae* coexisted, suggesting higher diversity than expected (Urban et al. 2024). One red squirrel strain, genetically distinct from modern variants, provides direct evidence of a historical animal reservoir—supporting a role for zoonoses in transmission ∼500 to 1,000 years ago (Pfrengle et al. 2021). In the Americas, leprosy has a second causative agent, *Mycobacterium lepromatosis*, recently detected in three ancient individuals dated to ∼335 to 665 years ago, highlighting the endemic presence of this pathogen in pre-Columbian populations (Lopopolo et al. 2025).

The *Mycobacterium tuberculosis* complex (MTBC) comprises at least nine lineages, thought to derive from environmental mycobacteria via genome reduction and specialization (Vargas et al. 2023; Guha et al. 2024). aDNA evidence reveals broad ecological dynamics, including zoonotic events. *M. pinnipedii*, typically infecting seals, was recovered from pre-Columbian Peruvian humans, showing inland spread from marine reservoirs (Bos et al. 2014; Vågene et al. 2022). Lineage 4 of *M. tuberculosis* has been identified in 250- to 370-year-old European remains, reflecting its long-standing global spread (Sabin et al. 2020; Jäger et al. 2022). Although most studies focus on MTBC sensu stricto, an ancient MAG of non-tuberculous mycobacteria (NTM) from the brain tissue of a mummy who died ∼238 years ago in Basel, Switzerland, shows that environmental species also occasionally infected humans in antiquity (Sarhan et al. 2023).

Paleogenomics has upended classic models of *Treponema pallidum* subsp. *pallidum*—the syphilis causal agent. A ∼2,000-year-old MAG from Brazil revealed *T. pallidum endemicum* (bejel), challenging the idea that syphilis arrived in Europe via the Columbian Exchange (Majander et al. 2024). Further, *T. pertenue* has been detected in ancient human remains from temperate regions of Mexico (>350 years ago), and Lithuania (350 to 430 years ago), contrasting with its current restriction to tropical zones (Barquera et al. 2020; Giffin et al. 2020). The discovery of a novel *Treponema* lineage in early modern Europeans further suggests a once broader treponemal diversity (Majander et al. 2024).

The recovery of a 600- to 700-year-old *Brucella melitensis* ancient MAG from the Western Mediterranean highlights long-term persistence of zoonotic pathogens (Long et al. 2023). Meanwhile, ancient dental calculus studies reveal the dynamics of oral microbiota. Although *Streptococcus mutans* are rarely found in ancient samples, MAGs from 4,000 years ago show early presence. Population expansions in *S. mutans* and *Tannerella forsythia* occurred ∼500 to 600 years ago—coinciding with increased sugar consumption—linking dietary shifts to microbial adaptation (Jackson et al. 2024). In addition, Neolithic remains (∼7,000 years ago) showed shifts in microbiome composition following the transition to agriculture, including a marked decline in *Methanobrevibacter* abundance, likely reflecting dietary changes (Quagliariello et al. 2022). The successful reconstruction of ancient *Borrelia* MAGs of 2,300 to 600 years ago provides insights into the evolution of vector-borne pathogens in Europe (Swali et al. 2025).

Ancient viral genomics has revealed complex patterns of emergence and persistence. In Eastern Eurasia, HBV genotypes B and D likely originated 5,000 to 400 years before present showed greater ancient diversity than today (Sun et al. 2024), with subgenotype shifts linked to past migrations. HSV-1 underwent a major lineage replacement around 4,700 years ago, challenging prior dispersal models (Guellil et al. 2022b). In contrast, variola virus (smallpox) shows evolutionary stability; ancient viral MAGs from around 600 to 1,000 years ago reveal gene loss absent in modern strains, suggesting long-term lineage coexistence (Bonczarowska et al. 2022).

Historical metagenomics has traced the emergence and spread of agricultural pathogens. The extinct HERB-1 haplotype of *P. infestans* dominated ∼182 years ago before replacement by US-1, like in Fig. 3 (Yoshida et al. 2013). Similarly, ancient *Xanthomonas citri* pv. *citri* (Xci), the cause of Asiatic citrus canker, likely diverged ∼11,500 years ago, spreading via early trade (Naqvi et al. 2022; Campos et al. 2023). Other non-human ancient pathogens reported were a viral genome of cassava mosaic virus (CMV) dated to ∼1849 (Chikoti and Tembo 2022) and the Equine Herpesvirus 4 (EHV-4) from a horse ∼1853 years ago, extending its evolutionary timeline by 3,800 years (Lebrasseur et al. 2024). Overall, while most of the focus has been on ancient human pathogens, the field is ripe for applications in a broader set of hosts.

## Expanding the Understanding of Pathogen Reservoirs

In wildlife, metagenomic studies have uncovered intricate viral ecosystems. For instance, bats frequently harbor co-circulating SARS-related and alpha-coronaviruses with coinfection rates up to 25%, creating hotspots for recombination and generating numerous novel viral genomes (Si et al. 2024). European shrews carry diverse novel paramyxoviruses and hepeviruses members phylogenetically close to human pathogens (Haring et al. 2024). Rodents in China's Yunnan Province, particularly *Rattus tanezumi*, are reservoirs for hantaviruses and lyssaviruses, especially in human-disturbed habitats (Kane et al. 2024). These findings underscore small mammals as critical sources of emerging viruses. Metagenomics is equally critical during outbreaks. During Uganda's 2022 Sudan virus outbreak, metagenomics revealed co-circulating pathogens including Crimean-Congo hemorrhagic fever virus strains not seen since 1958 (Balinandi et al. 2024).

Livestock represents another key interface for pathogen emergence. Shotgun metagenomics revealed a novel *Getah* virus variant in pigs from Guangdong, China, linked to severe disease (Chu et al. 2024). These insights emphasize the importance of ongoing genomic surveillance in animal populations to monitor emerging threats. Arthropod vectors also harbor diverse viral reservoirs. In Mexico, *Culicoides reevesi* midges carry seven novel viruses, including Nodaviridae and Totiviridae members (Laredo-Tiscareño et al. 2024). Ticks contain novel phlebovirus lineages and tick-borne viruses like Sulina virus and Tick-borne TCTV1 across Europe and Asia (Ergunay et al. 2024; Fang et al. 2024; Munjita et al. 2024).

Human-associated microbiomes are increasingly recognized as important reservoirs. A multidrug-resistant *Bacteroides cellulosilyticus* strain from bloodstream infection carried plasmid-encoded resistance and virulence genes, reflecting adaptability via HGT (Yin et al. 2024). Metagenomics enabled direct detection of *Campylobacter* sequence types and resistance genes from fecal samples with over 60% genome completeness (Djeghout et al. 2024). A large-scale study of 4,400 vaginal samples recovered over 18,000 prokaryotic genomes with relevant resistance profiles for women's health (Huang et al. 2024).

Beyond outbreaks and surveillance, shotgun metagenomics provides insights into pathogen evolution and adaptation. Influenza A surveillance in Saudi Arabia showed viral clade shifts and coinfections post-COVID-19, illustrating complex viral evolution and its impact on AMR (Dandachi et al. 2024). Soil metagenomics has identified diverse foodborne pathogens like *Listeria* species in US forest soils, shaped by altitude and soil properties (Wang et al. 2024).

Finally, plant-associated pathogens are emerging reservoirs of genomic innovation under environmental pressure. In *Capsicum* crops, *Xanthomonas perforans* exhibited strain shifts and increased genetic diversity under ozone stress, driven by both new mutations and selection on virulence variants (Kaur et al. 2025). Additionally, a novel RNA virus—Berkeley entomophthovirus—was discovered in the entomopathogenic fungus *Entomophthora muscae*, suggesting a role in host behavioral manipulation (Coyle et al. 2024).

## Evolution and Spread of AMR

AMR is a major global public health concern. Experimental evolution with pathogenic bacteria demonstrates rapid emergence of resistance via mobile genetic elements and mutations (Daruka et al. 2025). Functional metagenomics in *Escherichia coli* identified 66 previously unclassified AMR genes, many linked to stress responses (Suarez and Martiny 2024). Remarkably, nearly 40% of studied giant viruses (NCLDVs) carry AMR genes and virulence factors, positioning them as novel AMR reservoirs (Yi et al. 2024).

A major focus of recent microbiome research has been characterizing the spread and mobilization of AMR genes. Shotgun metagenomic studies consistently detect AMR genes in urban surface microbiomes across the World (Hartmann et al. 2016; Hsu et al. 2016; O’Hara et al. 2017; Fahimipour et al. 2018; Mahnert et al. 2019; Danko et al. 2021), and in a few cases, AMR genes have been associated with antimicrobial chemicals in indoors’ dust and surfaces (Hartmann et al. 2016; Fahimipour et al. 2018), implicating anthropogenic-driven selection on these systems. Additionally, extreme environments such as deep-sea cold seeps harbor vast resistomes with over 100,000 AMR genes, though high-risk variants remain rare (Zhang et al. 2025). Further, MAGs from ancient lineages from microbial mats in the Cuatro Ciénegas Basin revealed that AMR gene content in MAGs correlated with one of the major axes of variation (Rodríguez-Cruz et al. 2024). Thus, it is clear that AMR is not a recent phenomenon—nor one we are likely to eliminate. Instead, it has likely always spread through HGT. What has changed is the pace and scale of its dissemination under modern anthropogenic pressures.

## Microbial Domestication in Fermentative Environments

**Fig. 4.**
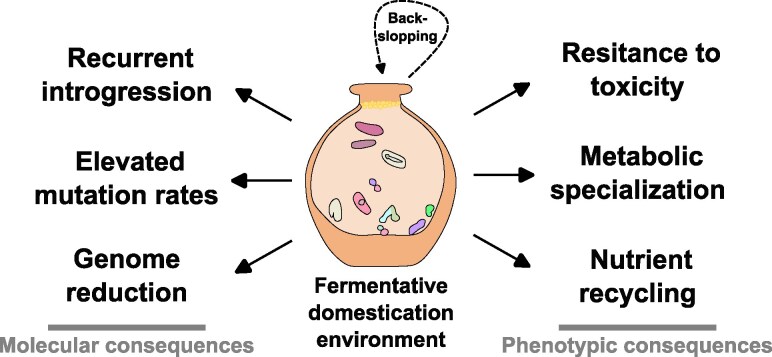
The consequences of microbial domestication in fermentative systems. Long-term human-mediated selection in fermentative environments, often maintained through practices such as back-slopping, exposes microbial communities to stable and reproducible conditions. Over time, this process drives genomic signatures of domestication (left), including recurrent introgression, elevated mutation rates and genome reduction, which in turn underpin phenotypic adaptations (right) such as resistance to toxicity, metabolic specialization, and nutrient recycling capacity.

Since the Neolithic period (∼13,000 years ago), human-driven domestication has shaped not only plants and animals but also, less visibly, microbial communities. A study of early human-managed systems found that the shift from natural ecosystems to controlled environments—such as fermenters, agricultural soils, and industrial bioreactors—has driven microbial divergence from their wild ancestors (Diamond 2002; Gibbons and Rinker 2015; Somerville et al. 2024). Fermentations represent one of the oldest forms of microbial domestication (Steensels et al. 2019; Leech et al. 2020; Somerville et al. 2024). Traditional fermentations rely on diverse and undefined microbial consortia selected for their metabolic capabilities (Paul Ross et al. 2002; Gibbons and Rinker 2015). In these systems, back-slopping—the reuse of inocula from previous fermentations—is a critical mechanism in shaping the evolution and adaptation of autochthonous microbial communities (Gibbons and Rinker 2015; Steensels et al. 2019). Within these environments, both bacterial and eukaryotic microorganisms undergo genome complex genomic reorganization, including structural and regulatory variation (Gibbons and Rinker 2015), as an adaptive response to the stability and resource richness of fermentative niches (Fig. 4). More recently, meta-analyses of shotgun metagenomics studies have found strong geographical signatures, as well as an abundance of AMR genes and several potential human pathogens among fermented foods (Xu et al. 2022; Liu et al. 2023). In modern meat fermentation, integrated metagenomics and metabolomics (see Box 2) found that starter inocula limits the growth of *Enterobacteriaceae*, likely by driving acidification and rapidly consuming fermentation substrates; however, the study also found increased consumer preference of spontaneous fermentation products, indicating a complex relationship between domestication and human consumption habits (Ferrocino et al. 2018). Overall, these studies indicate that the strong selective pressures of fermentative environments drive recurrent convergent metabolic specialization in microbes, which results in a pattern of similar functional profiles with a strong geographical signal.

Box 2. Metagenomics and technology development Metagenomics has dramatically expanded our understanding of biodiversity and enabled exploration of previously inaccessible environments. These breakthroughs have been driven by combined advances in sequencing technologies, sample processing, and computational tools. As a result, metagenomics has led the way in shaping methodological innovation across the Omic sciences—and this trend shows no signs of slowing.

To move beyond taxonomic profiling, researchers have turned to functional approaches such as metatranscriptomics (see Box 2). This actively captures the expressed genes in microbial communities, offering insights into their functional responses to environmental conditions (Aguiar-Pulido et al. 2016). However, its application remains challenging due to the large number of transcripts in a community (two orders of magnitude greater than the human genome) and the complexity of normalizing data across species abundance. Emerging tools, such as single-cell transcriptomics, could help overcome some limitations, although they introduce new difficulties. Spatial transcriptomics promises to resolve the physical structure and organization of communities, providing context to gene expression.

Deeper functional insights into microbial consortiums are provided by metaproteomics, which directly profiles protein synthesis. This enables the study of translational regulation, metabolic activity, and broader microbiota functions. Its application has increased in microbiology and the food industry, relying on mass spectrometry for protein detection and quantification (De Angelis and Calasso 2014; Vaccalluzzo et al. 2020; Archana et al. 2023). While significant challenges persist, especially in peptides characterization, ongoing technological innovation continues to push this field.

Meta-metabolomics complements other omics approaches by identifying community-derived metabolites, providing information on metabolic capacity and interspecies interactions (Bhosle et al. 2022). However, pinpointing the microbial source of individual metabolites remains a major obstacle. Techniques like nanoSIMS, which combine spatial resolution with single-cell precision, are increasingly used to address this issue—allowing, in some cases, for in situ measurements without disrupting the community structure.

Although cultivation predates metagenomics, the rise of *culturomics*—the large-scale cultivation of environmental microbes—has reinvigorated efforts to complement sequencing approaches. Culturomics advances media design, through genome-scale metabolic modeling, and isolation techniques, thereby enabling the recovery of rare or previously uncultured taxa and the assembly of synthetic communities for downstream applications (Matar and Bilen 2022; Clagnan et al. 2024).

In host-associated microbiomes, organoid systems—miniaturized, simplified versions of organs grown in vitro—have emerged as powerful models. These systems, pioneered in human microbiome research, allow for the controlled reconstruction of complex host–microbe interactions, providing invaluable mechanistic insights (Meirelles and Persat 2025).

The integration of these diverse data represents one of the most exciting frontiers in biological sciences. The combination of these approaches promises to validate and enrich metagenomic findings, offering deeper insights into the biological mechanisms, ecological interactions, and functional traits of complex microbiomes.

Ancient pathogen studies have transformed our view of disease evolution, showing that pathogens circulated earlier, more widely, and with greater diversity than once thought. These approaches reveal both long-term virulence stability and how human activities—like agriculture or medicine—drive microbial change. By going beyond traditional paleopathology, ancient DNA provides a more complete picture of historical disease dynamics and human-pathogen coevolution.

Metagenomic sequencing has revolutionized our understanding of pathogen reservoirs by enabling high-resolution detection, characterization, and surveillance of microbial communities across diverse hosts and environments. By revealing hidden viral diversity and complex ecological dynamics, these approaches are central to identifying spillover routes and assessing zoonotic potential.

Fermented agave-derived beverages—such as pulque and pre-distilled mezcal—represent well-documented model systems for studying microbiome domestication, with archaeological and historical evidence indicating that Mesoamerican cultures have maintained this practice for approximately 3,500 years (Lappe-Oliveras et al. 2008; Chacón-Vargas et al. 2020). Farmers have played an active role in this process by selecting agave plants with desirable fermentation traits, which over time has contributed to the co-domestication of associated microbial communities (Colón-González et al. 2025). Recently, shotgun metagenomic analysis of pulque fermentation revealed microbial responses associated with osmotic stress, toxin-antitoxin systems, and antiviral defense mechanisms. Further, phylogenomic reconstruction of *Saccharomyces cerevisiae* isolates from pulque showed close affinity to Asian isolates from ethanol-rich environments—suggesting convergent evolution across similar fermentative conditions (Chacón-Vargas et al. 2020). In parallel, a metabarcoding-based survey of mezcal fermentation sites across Mexico identified a diverse array of bacterial and fungal taxa (Jara-Servin et al. 2025). Notably, when compared with microbial communities from pulque, and despite regional differences in agave species, production practices, and environmental conditions, these studies identified a consistently shared core microbiome (Chacón-Vargas et al. 2020; Gallegos-Casillas et al. 2024; Kirchmayr et al. 2024; Jara-Servin et al. 2025). This indicates that long-term human influence has played a role in shaping microbial communities across geographically and culturally distinct fermentation systems (Fig. 4).

Due to its ease of cultivation and its central importance in fermentation processes, research on yeast domestication has primarily focused on large-scale isolation of individual strains followed by genome sequencing. These studies have revealed that domestication often results in both phenotypic and genomic divergence from wild populations (Fig. 4). A study on *S. cerevisiae*, *Torulaspora delbrueckii*, and *Brettanomyces bruxellensis* identified hallmark domestication traits in lineages associated with wine, bread, and dairy production (De Guidi et al. 2023). In *T. delbrueckii*, distinct functional signatures were found in isolates from different fermentation contexts. Wine-associated yeasts lacked functional aquaporins—likely an adaptation to osmotic stress—while those from dairy and bread environments showed enrichment in pathways related to galactose and maltose metabolism, respectively (Silva et al. 2023). Similarly, *S. cerevisiae* isolates from sourdough fermentations exhibited increased copy numbers of *MAL* loci, enhancing maltose utilization and supporting improved growth in carbohydrate-rich settings (Bigey et al. 2021). Collectively, these findings highlight how environment-specific selective pressures shape metabolic traits during the domestication of fermentative yeasts.

Although domestication signatures in bacteria are less well characterized than in yeasts, several compelling examples illustrate the genomic impact of adaptation to human-managed environments. In dairy fermentations, *Lactococcus* species have undergone extensive gene loss, particularly in amino acid biosynthesis pathways, as revealed by whole-genome sequencing. This metabolic reduction is compensated by a specialized protease system that breaks down casein, allowing these microbes to obtain essential amino acids directly from milk proteins. In addition, domesticated *Lactococcus* strains have also lost the ability to metabolize plant-derived sugars, reflecting a metabolic shift toward lactose as their primary carbon source (Siezen et al. 2005; Kelly et al. 2010). A similar pattern is observed in *Oenococcus oeni*, a species commonly associated with wine fermentations, whose genome exhibits notable reduction—likely linked to the loss of DNA repair mechanisms—leading to elevated mutation rates that may have accelerated its divergence from ancestral populations (Marcobal et al. 2008).

Overall, divergence between wild and domesticated microbes has been driven by selection for specialized metabolic traits that fulfill human needs, including food production, preservation, and biotechnological applications (Gibbons and Rinker 2015; Arias-Sánchez et al. 2019; Somerville et al. 2024). These patterns illustrate the long-term evolutionary impact of human selection on microbial genomes and highlight microbiomes as underappreciated targets of domestication (Fig. 4).

## The Evolutionary Consequences of Industrialization and Urbanization

Urbanization profoundly alters the natural environment. While cities have existed for millennia, urbanization has increased dramatically in recent decades (Seto et al. 2011). The ecological effects of urbanization on wildlife are well documented (Fischer et al. 2012), but their effect on microbes is less understood. aDNA metagenomics has revealed how zoonotic pathogens evolved in response to human practices (Fiddaman et al. 2023). In urban settings, pollution, humidity, UV exposure, antibiotic use, food sources, and surfaces create novel selective pressures, driving microbial adaptation in ways that differ from natural environments.

The impact of industrial pollution on microbial diversity is well-documented, mostly through metabarcoding approaches, revealing that different pollutants produce dramatic shifts on microbial communities (Wang et al. 2022). While many of the changes in diversity could be explained by selection acting on microbial species, the genetic mechanisms under selection have, in general, not been characterized. One shotgun metagenomic study of an industrial wastewater treatment facility, complemented by experimental validation, identified genes responsible for the biodegradation of common industrial contaminants (Pandit et al. 2021). Similarly, a study across three textile industrial wastewater plants identified various reductases, many with potential for bioremediation strategies (Kumar et al. 2021). Additionally, shotgun metagenomic characterization of sediment microbial communities across the Ganga river in India, a massive waterway which serves as an important environmental indicator, show that AMR profiles can be quite distinct despite similar taxonomic compositions (Rout et al. 2024a). These studies reinforce the idea that anthropogenic pollution directly exerts selective pressure on microbial communities, though critical gaps remain in quantifying the adaptive advantage conferred by these genetic elements, and on the environmental and health consequences of these adaptations.

Urbanization can also influence microbial evolution indirectly, by altering the behavior and diet of microbial hosts. Higher rates of HGT have been inferred in the human gut microbiota of urban westernized populations than in rural non-western populations (Groussin et al. 2021), though it remains unclear whether this pattern is specific to humans or reflects a broader urban-associated trend in host-microbiome evolution. A comparative study across four vertebrate species (including humans) along an urban-to-rural cline found that gut microbiotas of non-human species in urban environments became more similar to the human gut microbiota. The bacterial lineages enriched in urban wildlife paralleled those differentially abundant between rural and urban human microbiomes, suggesting convergent microbial adaptation to urban environments (Dillard et al. 2022). One likely explanation comes from another metagenomic study, coupled with stable isotope analysis, which showed that urban rodents develop an expanded dietary niche due to increased access to anthropogenic food sources (Anders et al. 2022). This overlap in dietary composition between urban wildlife and humans may facilitate recurrent microbial spillover across species, creating urban-specific microbiome transmission dynamics. Additionally, a recent focus on urban surface microbiomes has highlighted their role in AMR transmission (*see above*). However, low microbial biomass and high variability pose technical challenges for urban surface metagenomics, often resulting in low sequencing coverage. Recent developments of realistic in silico gold standards (Gerner et al. 2018) pave the way for future methodological advances. Overall, metagenomic studies have highlighted urban areas for their distinct evolutionary pressures, driven in part by antibiotic exposure, escalating pollution levels, and altered ecological interactions, though many other factors may play a significant role.

## Microbial Evolutionary Responses to Climate Change

At the functional level, there is a large body of work investigating the evolutionary response of single species to increased temperature, mostly through a combination of laboratory experiments and culture-based approaches. However, metagenomics has opened the door to investigate these processes in community context, mainly through experimental field studies. One such study tested the compounded effect of freeze-thaw winter cycles versus warming alone, and found that increased temperature suppressed bacteria with the genetic potential for carbon decomposition, nitrogen fixation and the final steps of denitrification; however, in the next season, communities that experienced a freeze-thaw cycle increased their predicted stress tolerance while reducing their predicted growth capacity, suggesting the emergence of an adaptive trade-off in response to freeze-thaw events (Garcia et al. 2020).

In another field study, reciprocal transplants across a climate gradient were paired with metagenomics and strain functional analysis, and found that trait variation among *Curtobacterium* ecotypes corresponds, at least partially, to local adaptation to climatic conditions (Chase et al. 2021). In a follow-up experiment, an isogenic *Curtobacterium* line was transplanted into all sites, and through a combination of metagenomic sequencing, and re-isolation and genome sequencing the authors found candidate de novo mutations for environmental adaptation, including a variant in an exopolysaccharide-producing enzyme that may be adaptive in extreme drought and high temperatures. However, no selective sweeps were detected, which probably reflected the very low estimated effective population sizes (10^2^ to 10^3^) and small estimated number of generations (10^3^) (Chase et al. 2021).

Another glimpse on the potential consequences of climate change comes from observational studies on glacial melt and runoff. Metagenomic characterization of lake sediments across heterogeneous runoff regimes in an arctic lake found that increased runoff correlates with reduced diversity and metabolic potential of its dominant microbial communities (Colby et al. 2020). Further, MAGs derived from the same lake displayed a high prevalence of pathways involved in lipid chemistry, and a low prevalence of nutrient uptake pathways, suggesting a high degree of local adaptation to the low temperatures, and oligotrophic conditions of the site (Ruuskanen et al. 2020). It is unclear how these highly adapted lineages would fare through the ongoing consequences of climate change, and it remains a pressing question to determine what would be the direction and magnitude of the changes of these microbial communities on biogeochemical cycles (Colby et al. 2020).

Climate change has also been predicted to increase the range of animal and plant pathogens (IPPC Secretariat 2021; Geng et al. 2025), likely increasing disease rates across the World. Additionally, shifting pathogen ranges may also increase the risk that a pathogen infects a new type of host, but this spillover risk is difficult to assess. By combining shotgun metagenomics and metatranscriptomics (see Box 2), a recent study reconstructed the virosphere and viral host range across a range of glacial runoff conditions; the authors then compared the congruence between the viral and host phylogenies on each site, under the assumption that weaker congruence is a marker of increased spillover risk. The authors found that increased runoff correlated with decreased phylogenetic congruence in lake sediments, but not in soils (Colby et al. 2020). While these spillover risk measurements remain indirect, this study shows the power of shotgun metagenomics to quantitatively investigate some of the potential consequences of climate change. Larger and more systematic studies of this type are urgently needed. Ultimately, understanding the microbial evolutionary response to climate change will require integrating dynamics at multiple levels of biological organization, from strains to communities (Martiny et al. 2023), a challenge for which metagenomics is uniquely well suited.

## Future Directions and Challenges

Microorganisms have been fundamental drivers of life on Earth, shaping its history from the atmospheric modification events billions of years ago, to daily transmission dynamics, and into the consequences of anthropogenic-driven climate change. Metagenomics has revealed new branches of the Tree of Life and provided molecular details on ancient events. Despite advances, the timing of specific, ancient events such as the position of the root in the Tree of Life, and the branching order of deep lineages remains unclear. In this regard, the choice of substitution models has a notable effect on the phylogenetic reconstruction, and no method can fully account for long-branch attraction (Gouy et al. 2015). Recent work using manually curated marker sets has highlighted the importance of detecting inter-domain HGT and hidden paralogy, as well as accounting for substitutional saturation, which can lead to underestimated divergence times in deep branches, particularly in the archaea-bacteria split (Moody et al. 2022).

The increasing accessibility of aDNA has significantly expanded our ability to investigate life's past. Metagenomic analysis of aDNA enables ancestral environmental reconstructions and allows researchers to trace the evolutionary history of microbial lineages. While much of this work has focused on pathogens, there is growing potential to explore the evolution of environmental microbes, particularly those preserved in permafrost and melting glaciers. However, most existing bioinformatics tools were originally developed for modern DNA and may not perform optimally on degraded aDNA. Although some tools have shown promise in recovering ancient MAGs from simulated aDNA datasets, their performance on real archaeological samples remains challenging due to the higher complexity and degradation of authentic ancient material (Standeven et al. 2024).

Microorganisms play a central role in Earth's biogeochemical processes. By integrating methods from environmental DNA analysis, molecular biology, microbiology, organic geochemistry, and the geological record, geobiology offers powerful tools to reconstruct the evolution of life and Earth over deep time (Fluegeman 2014). Across extant ecosystems, the boundaries of life on Earth continue to be redefined as metagenomics leads to the discovery of microorganisms thriving in extreme conditions, such as hydrothermal vents, hyperalkaline lakes, volcanic environments, and polar ice caps. Metagenomics has become a powerful tool for investigating the physiology, ecology, and adaptations of microorganisms inhabiting these environments. By bypassing the limitations of cultivation-based methods, metagenomic approaches provide unprecedented insights into the functional potential, adaptive strategies, and the limits of life (Gutiérrez-Preciado et al. 2024; Zhou et al. 2025).

Beyond their ecological roles, microorganisms have profoundly influenced macroorganismal evolutionary trajectories across geological times, through plagues, pandemics, and other diseases. Metagenomics has significantly advanced our understanding of host-associated microbiota evolution, but key challenges remain in distinguishing adaptive evolution from ecological turnover, resolving microbial transmission patterns, identifying selection signals, and integrating functional validation. While environmental metagenomics provides an invaluable genomic snapshot of host-associated microbiomes, the integration of culturomics—high-throughput microbial cultivation combined with genomic characterization—has emerged as a powerful complementary approach in multiple host and non-host systems (Lundberg et al. 2022; De Guidi et al. 2023; Baker et al. 2025).

Human activities have been shaping microbial evolution since our origins. The domestication of microorganisms enabled civilizations to achieve remarkable advancements in agriculture, fermentation, and industrial processes. Microbial domestication has driven diversification and innovation across societies, highlighting the potential of microbes as tools for human progress. However, the Anthropocene has introduced new challenges by creating novel selection pressures that reshape microbial communities. Rising temperatures, pollution, urban agglomeration, and other environmental stressors are potentiating processes such as HGT transfer and the spread of AMR genes.

Ultimately, the evolutionary consequences of climate change, and other anthropogenic pressures, on microbial ecosystems remain an open and pressing question. Experimental evolution and field studies suggest that microbial populations can adapt rapidly to new thermal environments, but such adaptations may come at the cost of reduced evolutionary potential or increased sensitivity to future perturbations (McGaughran et al. 2021). Moreover, the eco-evolutionary dynamics in these ecosystems are not only shaped by temperature alone but by complex interactions between host organisms, nutrient availability, and shifting microbial community structures (Martiny et al. 2023). Moving forward, a major challenge will be linking observed genomic shifts to geoecosystem-level processes such as carbon cycling, greenhouse gas emissions, and pathogen emergence. Addressing these challenges will require leveraging longitudinal metagenomics, experimental evolution, microcosm reconstitution experiments, functional assays, and predictive modeling to establish the evolutionary mechanisms shaping host–microbe and microbe–microbe interactions with the environment.

In this review, we highlighted how metagenomics has revolutionized our understanding of microbial life, from deep branching events—billions of years ago—to the complex microbiomes of modern urban civilizations. This powerful approach allows us to trace the profound influence of microorganisms over billions of years, revealing how they have shaped and modulated life on Earth (Fig. 1). Such insights have been made possible by rapid advances in genomic technologies. However, significant knowledge gaps and opportunities remain in many ecosystems, underscoring the urgent need for further research—especially to understand microbial responses to climate change and the increasing human impact.

## Data Availability

No data was generated for this manuscript. All references and materials are available from the corresponding publishers.

## Glossary

Adaptation: A heritable trait that increases the fitness of an organism in a given environment, arising through natural selection.
Ancient DNA (aDNA): DNA retrieved from archaeological, paleontological, or historical biological materials, such as bones, teeth, hair, mummified tissues, coprolites, sediments, or permafrost, from organisms that lived in the past. Often degraded and fragmented due to age.
Antimicrobial resistance (AMR): The ability of microorganisms to survive and grow despite exposure to drugs that would normally inhibit or kill them, such as clinical antibiotics.
Commensal: A microorganism that lives in or on a host without causing harm.
Domestication: The process by which humans shape the evolution of other organisms, such as microbes, through artificial selective pressures in managed environments (e.g., agriculture, fermentation).
Haplotype: A set of genetic variants or alleles inherited from a single parent.
Horizontal gene transfer (HGT): The movement of genetic material between unrelated individuals.
Linkage disequilibrium (LD): The nonrandom association of alleles at two or more loci in a population, such that certain allele combinations occur more or less frequently than expected.
Maar lake: A lake formed by groundwater or precipitation filling a volcanic crater.
Macroevolution: Large-scale evolutionary change, occurring at or above the species level.
Metagenome-assembled genome (MAG): An approximation of an organism’s genome. A collection contigs which can be grouped (binned), typically based on a combination of nucleotide composition, co-occurrence, and physical proximity, and thus are likely to derive from the genomes of highly related individuals.
Metagenomics: The study of all genetic material in an environmental sample. Typically characterized by sequencing via metabarcoding, which focuses on markers such as rRNA genes, or shotgun metagenomics, which attempts to sequence all the DNA of the sample.
Microevolution: Small-scale evolutionary changes within populations, occurring below the species level.
Oligo-colonization model: A scenario in which different microbial strains coexist at intermediate frequencies within a host, with strain identity and abundance varying across individuals.
Oligotrophic environment: An ecosystem with low nutrient availability, often favoring microorganisms adapted to resource scarcity.
Panselectionism: The view that evolutionary change results almost entirely from natural selection acting on genetic variation, with little role for neutral processes.
Pathogen: A microorganism capable of causing disease in a host.
Priority effects: Ecological outcomes determined by the order and timing of species arrival.
Phylosymbiosis: The pattern in which similarities among microbial communities reflect the phylogenetic relationships of their hosts.
Relative evolutionary rate (rER): A comparative measure of the speed at which genetic changes accumulate in different lineages or genes in comparison to a reference, often the genome-wide or background rate within a group of species.
Site frequency spectrum (SFS): A summary statistic in population genetics that describes the distribution of allele frequencies across sites within a population.
Virulence factor: A genetic trait or molecule that enhances a pathogen’s ability to infect, cause disease, or evade the host immune system.
